# Alteration of Interneuron Immunoreactivity and Autophagic Activity in Rat Hippocampus after Single High-Dose Whole-Brain Irradiation

**DOI:** 10.7759/cureus.1414

**Published:** 2017-06-30

**Authors:** Yi-Bing Ouyang, Shoucheng Ning, John R. Adler, Bruce Maciver, Susan J Knox, Rona Giffard

**Affiliations:** 1 Anesthesia, Stanford University School of Medicine; 2 Department of Radiation Oncology, Stanford University Medical Center; 3 Department of Neurosurgery, Stanford University School of Medicine

**Keywords:** whole-brain radiation, interneurons, hippocampus, autophagy, parvalbumin, calbindin, reelin, neuropeptide y

## Abstract

The effects of high dose gamma radiation on brain tissue are poorly understood, with both limited and major changes reported. The present study compared the effects of gamma irradiation on the expression of interneuron markers within the hippocampal cornu ammonis 1 (CA1) region with expression in control matched rats. This area was chosen for study because of its well-characterized circuitry. Male Sprague-Dawley rats were exposed to 60 Gy of whole brain gamma radiation and after 24 or 48 hours, the brains were removed, fixed and sectioned to quantitate expression of parvalbumin (PV), calbindin-D28K (CB), reelin, neuropeptide-Y (NPY), and somatostatin. All of these markers increased in expression over the first 48 hours, except NPY, which decreased. This provides novel information on changes in gene expression in the hippocampal interneurons following radiation. Staining for Beclin 1, a marker of autophagy, increased most strongly in the subgranular zone (SGZ) of the dentate gyrus (DG). Overall, the results are consistent with the hypothesis that increased intracellular calcium follows irradiation, leading to an increased expression of calcium binding proteins. Increased autophagy occurs in the neurogenic zone of the dentate hilus, consistent with reduced effective neurogenesis after irradiation.

## Introduction

Whole-brain irradiation (WBI) and stereotactic radiosurgery (SRS) have been used for many years for the treatment of primary brain tumors and central nervous system (CNS) metastases. More recently, SRS has begun to be explored as a treatment modality in a variety of pain, psychiatric, and movement disorders. However, the mechanism of action in these diseases is not understood, nor have their potential complications been well studied. For example, SRS with high dose photons is currently being explored as a treatment for severe depression [1-2]. For this study, a high dose of WBI was chosen as this is relevant to stereotactic doses of radiation used, for example, to treat trigeminal neuralgia [3]. Although depression is a highly disabling and phenotypically heterogeneous psychiatric disease, affecting up to 17% of the worldwide population at least once in their lives, the underlying mechanism(s) is poorly understood [4]. It is reported that a major depressive disorder is associated with impaired function of certain subtypes of gamma-aminobutyric acid-ergic (GABAergic) interneurons [5-6]. However, to our knowledge, there have not been any studies on the effect of radiation on interneurons. We chose seven different interneuron markers to study this immunohistochemically, and it is presented in Table 1. Prior studies have shown different types of damage after brain irradiation including effects on neural structure, plasticity, and neurogenesis [7]. One undesirable side effect of WBI is long-lasting depression of neurogenesis in the hilus of the dentate gyrus (DG) which significantly affects the hippocampal function [8]. Because of the important regulatory role played by interneurons in the hippocampal circuitry, as well as their role in the generation of gamma and theta rhythms, the present study investigates radiation-induced changes of immunohistochemical markers for interneurons and autophagy in rat hippocampus after a single high-dose WBI [9-10].

## Materials and methods

Rat Brain Radiation

All experiments were performed according to the protocol approved by the Stanford University Administrative Panel for Laboratory Animal Care. Male Sprague Dawley rats, 26 days old and 80-100 grams in body weight, were purchased from Charles River Laboratories. The rats were maintained under pathogen-free conditions, and sterilized food and water were available ad libitum. The rats were randomly assigned to two groups: sham irradiation control and 60 Gy ionizing gamma radiation. The rats were anesthetized with an intraperitoneal injection of a cocktail of ketamine (70 mg/kg) and xylazine (7 mg/kg) immediately before irradiation. The anesthetized rats were then placed in individual lead boxes with the upper part of the head protruding through a cutout window at the front of each box. Radiation was delivered using a Philips RT-250 200 kVp X-ray unit (12.5 mA; half-value layer, 1.0 mm Cu) at a dose rate of 140 cGy/min. The entire animal brain was locally irradiated with a single dose of 60 Gy. After irradiation, the rats were returned to their cage for recovery.

Immunohistochemistry

Irradiated or sham-operated rats were deeply anesthetized and perfused transcardially with cold 0.9% saline, followed by 4% paraformaldehyde in phosphate-buffered saline (PBS) pH 7.4, 24 or 48 hours after 60 Gy gamma irradiation, n=6 in each group. The brains were kept in 4% paraformaldehyde in PBS for 3 days then cut into 50 μm coronal sections with a vibratome (VT1000S, Leica Microsystems, Wetzlar, Germany). Free-floating sections were washed in PBS and then treated with 1% H_2_O_2_ for 20 minutes. Nonspecific binding was prevented by incubating the sections for one hour in 5% normal goat serum in PBS containing 0.3% triton X-100. The sections were incubated overnight at 4^◦^C with various primary antibodies (Table 1). After washing it three times in PBS, the sections were then incubated for an hour at room temperature in a secondary antibody solution made up in PBS (Table 1). The sections were washed and mounted on slides with Vectashield mounting medium for fluorescence in some cases containing 4',6-diamidino-2-phenylindol (DAPI) (Vector Laboratories, Inc. Burlingame, CA) and studied by epifluorescence microscopy using a Nikon Axiovert LSM510 (Carl Zeiss, Goettingen, Germany).

**Table 1:** Primary Antibodies

Primary Antibodies				
Primary Antibody	Marker	Dilution	Source	Host
Anti-Parvalbumin	Interneuron	1:5000	SWANT #: 235	Mouse
Anti-Calbindin D-28K	Interneuron	1:5000	SWANT #: 300	Mouse
Anti-Reelin	Interneuron	1:5000	EMD Millipore #: MAB5364	Mouse
Anti-NeuN	All Neurons	1:500	EMD Millipore #: MAB377	Mouse
Anti-Neuropeptide Y	Interneuron	1:500	Abcam #: ab30914	Rabbit
Anti-Somatostatin-14	Interneuron	1:5000	Bachem Peninsula #: T-4103	Rabbit
Anti-Cholecystokinin	Interneuron	1:500	Sigma-Aldrich #: SAB2100357	Rabbit
Anti-nNOS	Interneuron	1:500	Abcam #: ab1376	Goat
Anti-Beclin 1/ATG6	Autophagy	1:200	NOVUS NB500-249	Rabbit

**Table 2:** Secondary Antibodies

Secondary Antibodies		
Secondary Antibody	Dilution	Source
Donkey anti-Rabbit IgG (H+L), Alexa Fluor® 594	1:200	Life Technologies #A-21207
Donkey anti-Mouse IgG (H+L), Alexa Fluor® 594	1:20	Life Technologies #A-21203
Donkey anti-Goat IgG (H+L), Alexa Fluor® 594	1:200	Invitrogen #A-11058
Donkey anti-Rabbit IgG (H+L), Alexa Fluor® 488	1:200	Invitrogen #A-21206

Quantitation and Statistical Analysis

**Figure 1:**
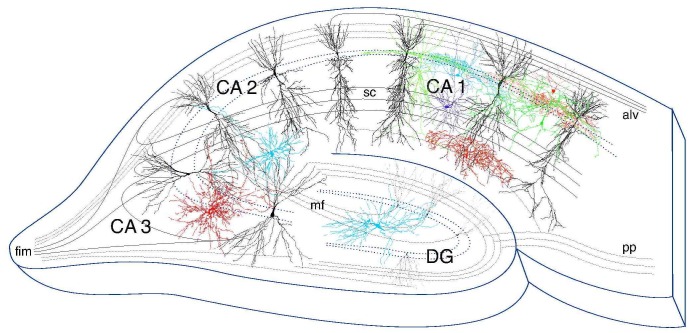
Diagram of the hippocampus showing subregions CA1: cornu ammonis region 1; CA2: cornu ammonis region 2; CA3: cornu ammonis region 3; DG: dentate gyrus; fim: fimbria pathway; mf: mossy fiber pathway; sc: Schaffer collateral pathway; alv: alveus; pp: perforant pathway axons.

**Figure 2:**
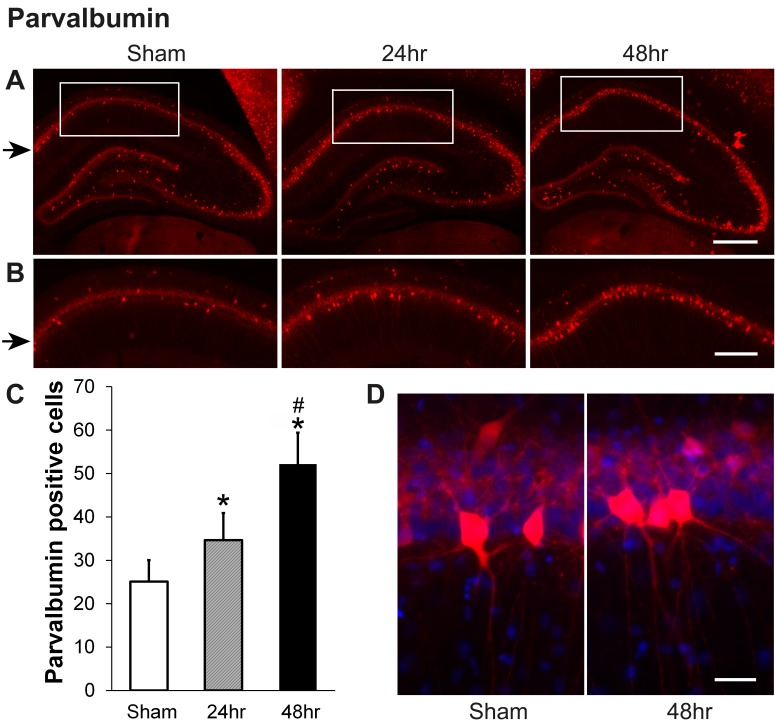
Parvalbumin (PV) increases in hippocampal CA1 area at 24-hour and 48-hour recovery after a single dose of 60 Gy gamma radiation (A) Representative images showing PV staining (red color) in the hippocampus from sham, and after 24-hours and 48-hours recovery following irradiation. (B) Higher power views of hippocampal CA1 (boxes in A) indicate where the PV-positive neurons were counted. (C) Quantification of PV-positive neurons in hippocampal cornu ammonis 1 (CA1) area shown at higher magnification in panel B; N=6 rats/group x 5 slices/animal, P<0.01 compared to sham (*) or to 24 hours (#). (D) Clusters of PV-positive neurons were observed in the 48-hour recovery group but not in sham; black arrows indicate the CA1 layers. Scale bars: A, 400 µM; B, 200 µM; D, 25 µM.

**Figure 3:**
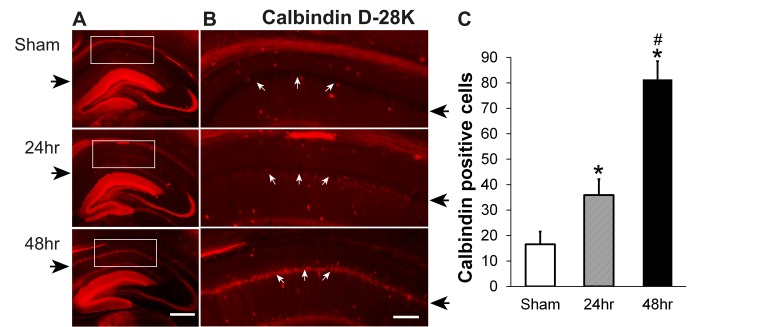
Calbindin D-28K (CB) increases in the hippocampal CA1 area 24 hours and 48 hours after a single dose of 60 Gy gamma radiation (A) Representative images showing CB staining (red color) in the hippocampus from sham, and rat brains 24 hours and 48 hours after irradiation. (B) Higher power views of hippocampal CA1 (boxes in A) indicate where the CB-positive neurons were counted; arrows show the hippocampal CA1 pyramidal layer. (C) Quantification of CB-positive neurons in the hippocampal CA1 area shown in B; N=6 rats/group x 5 slices/animal, P<0.01 compared to sham (*) or to 24 hours (#); Black arrows indicate CA1 layers. Scale bars: A, 400 µM; B, 120 µM.

**Figure 4:**
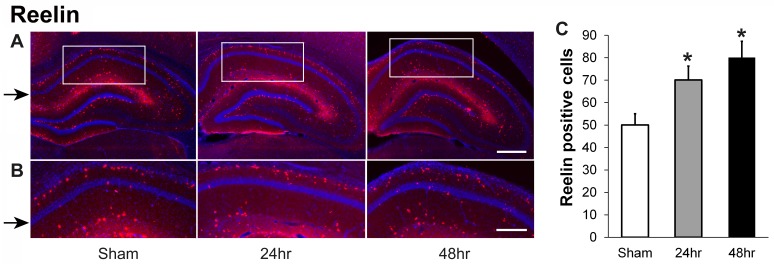
Reelin-positive cells increase in the hippocampal CA1 area 24-hours and 48-hours recovery after a single dose of 60 Gy gamma radiation (A) Representative images showing reelin staining (red color) in hippocampus from sham, and after 24-hours and 48-hours recovery following irradiation; cell nuclei are stained with 4’6-diamidino-2-phenylindol (DAPI) (blue). (B) Higher power views of hippocampal CA1 (boxes in A) indicate the areas in which reelin-positive neurons were counted. (C) Quantification of reelin-positive neurons in CA1 within the area shown in panel B; N=6 rats/group x 5 slices/animal, P<0.01 compared to sham (*); black arrows indicate CA1 layers. Scale bars: A, 400 µM; B, 200 µM.

**Figure 5:**
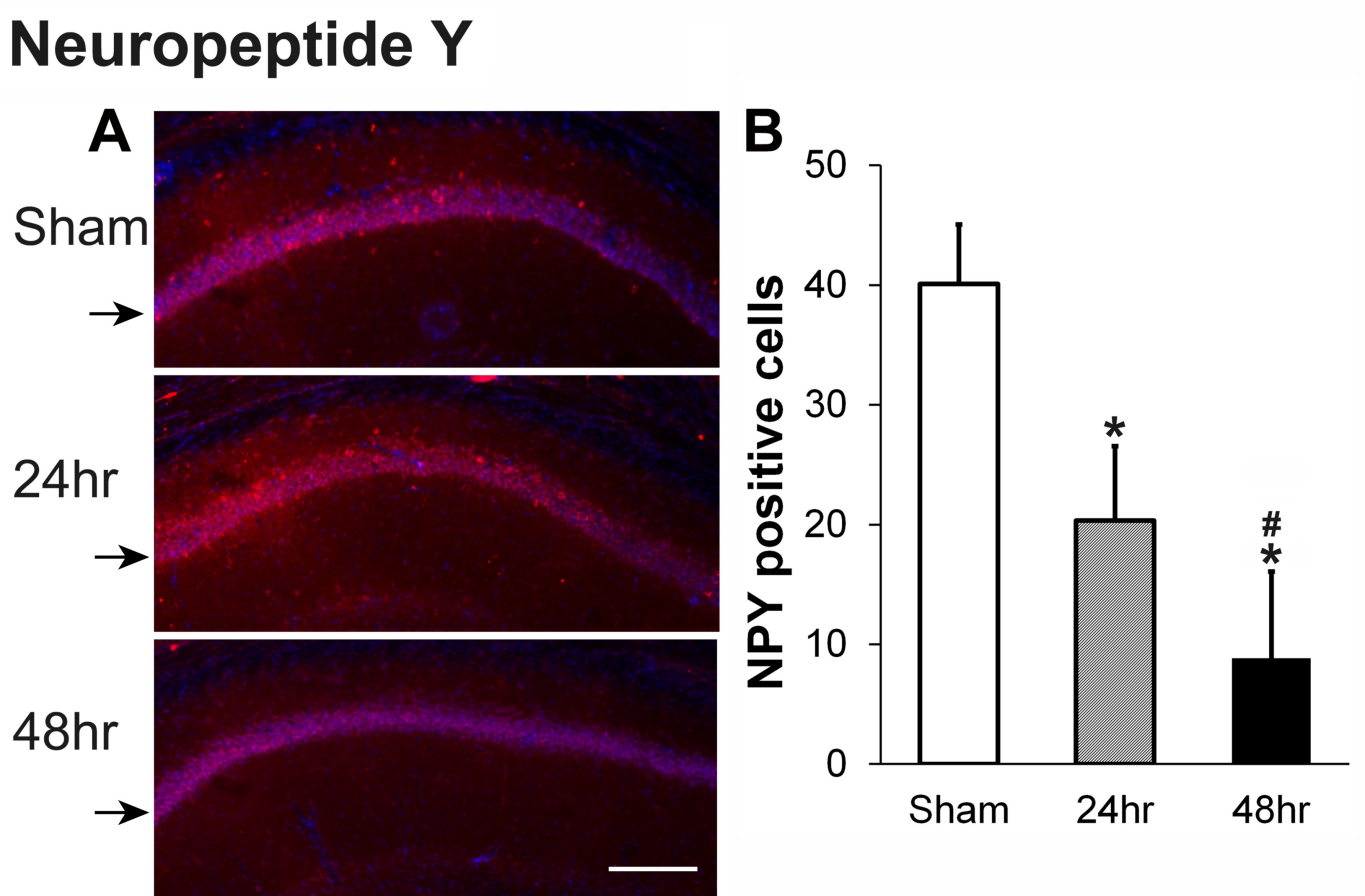
Neuropeptide Y (NPY) decreases in the hippocampal CA1 at 24 hours and 48 hours after a single dose of 60 Gy gamma radiation (A) Representative images showing NPY staining (red color) in the hippocampal CA1 area from sham, and brain harvested 24 hours and 48 hours after irradiation; cell nuclei are stained with DAPI (blue). (B) Quantification of NPY-positive neurons in the hippocampal CA1 area illustrated in A; N=6 rats/group x 5 slices/animal, P<0.01 compared to sham (*) or to 24 hours (#); black arrows indicate CA1 layers. Scale bar: 200 µM.

**Figure 6:**
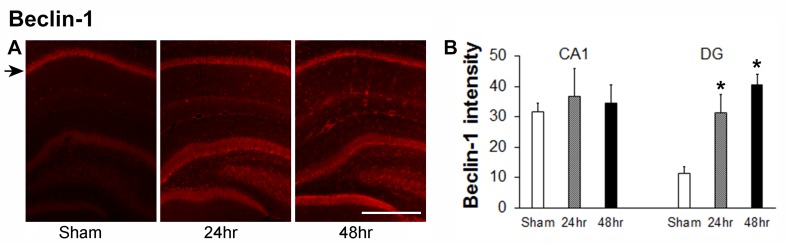
Changes of beclin 1 staining in hippocampal CA1 and DG areas 24 hours and 48 hours after a single dose of 60 Gy gamma radiation (A) Representative images showing beclin 1 staining in the hippocampus from sham, and brains harvested 24 hours and 48 hours after irradiation. (B) Quantification of beclin-1 immunoreactivity in hippocampal CA1 and dentate gyrus (DG) areas showed in A; average intensity was measured in 6 rats/group x 3 slices/animal, P<0.01 compared to sham (*); black arrows indicate CA1 layers. Scale bar: 400 µM.

A diagram of the hippocampus with subregions indicated is shown in Figure 1. The numbers of immunoreactive interneurons were counted in the hippocampal cornu ammonis 1 (CA1) region as shown in Figure 1, and in a fixed area as shown in Figures 2-5. Five slices/animal for each of the six animals/group were counted. In Figure 6, quantitation of fluorescence staining intensity of beclin 1 positive neurons was performed by selecting a fixed area in the hippocampal CA1 or the DG areas using the Lasso tool and taking the intensity numbers from the histogram in Adobe Photoshop CS6. The average intensity was measured in three slices/animal for six rats/group. The data reported are means ± SD. The statistical difference was determined using ANOVA followed by Newman Keuls posttest. P < 0.05 was considered significant.

## Results

Parvalbumin (PV) increased 24 hours and 48 hours after irradiation. In the sham and irradiated groups, PV-specific immunoreactivity was mainly detected in the nonpyramidal neurons of the hippocampal CA1-3 region and hilar neurons of the DG. PV-positive fibers were also detected in almost all regions of the hippocampus (Figure 2). Some differences were noted between the sham and irradiated groups. We counted PV-positive neurons in the hippocampal CA1 area within the area marked (Figure 2A). In the CA1 region, the number of PV-positive neurons was significantly increased in the 24-hour and further in the 48-hour group (Figures 2B-2C). Interestingly, clusters of PV-positive neurons were found in the 24 and 48-hour groups but not in the sham group of animals where PV-positive cells were generally spaced further apart (Figure 2D).

Calbindin-D28K (CB) also increased progressively 24 hours and 48 hours after irradiation (Figure 3A-3C). We counted CB-positive neurons in the indicated area within the hippocampal CA1 region (Figure 3A). In the control and irradiated groups, CB-specific immunoreactivity was strongest in the granule cell layer of the DG and mossy fibers. There were very few CB-positive cells in the hippocampal CA1 region of the sham group (Figures 3A-3B, upper panels) while irradiation treatment significantly increased the number of CB-positive cells in the CA1 region at 24 hours (Figures 3A-3B, middle panels) and quite markedly at 48 hours of recovery (Figures 3A-3B, lower panels). Quantitation is shown in Figure 3C.

Reelin-immunoreactive cells also increased at 24 hours and 48 hours after irradiation. Reelin-positive neurons, observed largely on the upper border of the hippocampal CA1 pyramidal layer, were counted within the marked rectangular area (Figure 4A). There are few reelin-positive cells near the pyramidal neurons of the CA1 region in all groups (Figure 4B). Although increased numbers of reelin-positive cells were observed as seen in Figure 4C, smaller dots possibly representing cell fragments were observed only in the 24 and 48-hour groups (Figure 4B, middle and right panels).

Of the interneuron markers studied here, only neuropeptide-Y (NPY) immunoreactive cells decreased 24 hours and 48 hours after irradiation. As shown in Figure 5A, in the sham rat brain, NPY-positive neurons exist close to pyramidal neurons of the hippocampal CA1 region. However, after irradiation, the number of NPY-positive interneurons decreased significantly compared with the sham group, with few NPY-positive neurons left in the 48-hour recovery group (Figure 5B).

If radiation causes cell impairment, there might be an evidence of increased autophagy. To assess this, we stained for beclin 1. Beclin 1 immunoreactivity after irradiation increased markedly, but only in the DG. Staining in the CA1 area did not differ much between groups. However, the intensity of beclin 1 staining increased in the DG area, especially the subgranular zone (SGZ) of the DG, where the neurogenic niche is located, 24 and 48 hours after irradiation (Figure 6A, middle and right panels). Quantitation of beclin 1 staining intensity in CA1 and the DG shows the marked increase only in the DG (Figure 6B). We performed similar staining for cholecystokinin, somatostatin, and nitric oxide synthase (nNOS) but these were not quantified because of weak staining. Terminal deoxynucleotidyl transferase dUTP nick end labeling (TUNEL) staining revealed no clear evidence of apoptosis at either 24 or 48 hours after irradiation.

## Discussion

Brain irradiation has been reported to induce many acute and chronic changes at the cellular and molecular level [7]. For the first time, we demonstrate here that in the early phase (24 and 48 hours) after a single 60 Gy gamma-ray whole-brain exposure, there are significant changes in markers for interneurons and autophagy in rat hippocampus.

Two of the major types of neurons are glutamatergic principal neurons and GABAergic interneurons. In terms of neuron numbers, although GABAergic cells represent a minority, they serve important functions. A hallmark of interneurons is their structural and functional diversity. In the CA1 region of the hippocampus alone, 21 different classes of interneurons have been distinguished [11].

PV is a small (typically 9-11 kDa) stable protein containing EF-hand type calcium binding sites and is involved in calcium signaling. PV interneurons have a causal role in the cortical gamma oscillation generation [9-10]. PV-containing interneurons are fast spiking, and generate and maintain gamma oscillations necessary for attention, cognitive flexibility, and performance [12]. Hippocampal interneurons receive both internal and external input. Recently, it was demonstrated that PV-containing interneurons, in the hippocampus, are the key components of intrinsic hippocampal theta oscillators [13]. Alterations in the function of the PV-expressing neurons have been implicated in Alzheimer’s disease (AD) [14]. During the early period after ischemia-reperfusion (from 30 minutes to three hours), there was a rapid but transient increase in the number of PV-positive neurons in the parietofrontal cortex, explained as an increase in intracellular calcium concentration [15]. To our knowledge, the current study is the first report that shows a PV expression increase after radiation and suggests this could reflect changes in the intracellular calcium subsequent to irradiation.

CB is a calcium buffering and calcium sensing protein which contains four active calcium-binding domains. A reduced density of CB-immunoreactive GABAergic neurons was observed in the occipital cortex during major depression [6]. In the present study, the increased number of CB expressing neurons may also indicate increased intracellular calcium concentration, consistent with the increased PV expression.

Reelin is a large extracellular matrix glycoprotein important for proper neuronal migration during brain development due to its participation in cell-cell interactions. Besides this important role in development, reelin also has functions in the adult brain. It modulates synaptic plasticity by enhancing the induction and maintenance of long-term potentiation and stimulates dendrites and dendritic spine development [16-17]. It regulates the continuing migration of neuroblasts generated in the subventricular and SGZ, the sites of adult neurogenesis. Reelin levels were decreased in the post-mortem hippocampal tissue from patients with schizophrenia, bipolar disorder, and depression, and also in an animal model of depression [18].

Reelin may also play a role in AD. A decrease of reelin expression in the hippocampus occurs during normal aging and is associated with cognitive decline and with the appearance of amyloid β (Aβ) plaques [19-21] Moreover, a recent study demonstrated that intracellular expression of Aβ in layer II of the entorhinal cortex is limited to reelin-immunoreactive neurons of AD patients and in a rat AD model, during the pre-plaque stage, suggesting a physical interaction between reelin and intracellular Aβ [21]. Our results indicate an increase in the numbers of reelin-positive neurons after radiation, the meaning of which is not clear.

NPY is a neuropeptide neurotransmitter in both the brain and the autonomic nervous system which has roles in stress, food intake, obesity, and seizures [22]. The decrease of NPY-positive neurons in this study agrees with results of the electrophysiology study we published which used the same radiation protocol in the same age and strain of rat [23].

Autophagy is a highly conserved and regulated process of lysosomal degradation of organelles and macromolecules. Autophagy is required for all cells to maintain cellular homeostasis. However, it can be both protective against stress and contribute to brain injury in some settings [24-25]. In cancer research, autophagy significantly contributes to the effects of radiation [26]. The increased beclin 1 expression seen here in the hippocampal SGZ of the DG might represent a side effect of high-dose irradiation, likely related to decreased effective neurogenesis. Decreased hippocampal neurogenesis was reported in young rats after fractionated 10 Gy WBI and irradiation can induce long-lasting declines in cognitive function in adults who received radiation as children [27-29]. The pattern of beclin 1 expression is consistent with an increase in failed neurogenesis and is likely related to effects including persistent inflammation, as observed earlier by others [30].

In summary, 60 Gy whole brain radiation in rats increased expression in CA1 of three interneuronal markers (PV, CB, and reelin) while decreasing expression of NPY. Because we observed marked changes in the expression over a short period of time, 48 hours, this is unlikely to represent new neurons, but rather a change in gene expression in neurons within the CA1 region. It is unclear whether these changes in the interneurons play a role in either the beneficial or deleterious effects of radiation in the CA1 region, and merit further investigation to elucidate the potential clinical significance of these observations.

## Conclusions

Two interneuron markers were involved in the calcium homeostasis increase in the CA1 hippocampal subregion during the first two days following whole brain radiation, suggesting a possible response to calcium changes. The increase observed in reelin could be associated with plastic changes induced by radiation. The one neurotransmitter assessed, NPY, was the only marker that decreased. Evidence of increased autophagy was seen in DG but not CA1. Overall, all the interneuron markers assessed, changed within 48 hours following 60 Gy whole brain radiation. More studies are needed to better understand the effects of radiation on interneurons and to characterize the interaction of interneurons after radiation with other cells in the brain.
